# Polymer composite microspheres loading ^177^Lu radionuclide for interventional radioembolization therapy and real-time SPECT imaging of hepatic cancer

**DOI:** 10.1186/s40824-023-00455-x

**Published:** 2023-11-04

**Authors:** Liu Xiao, Yuhao Li, Ruiman Geng, Lihong Chen, Peng Yang, Mingyu Li, Xia Luo, Yuchuan Yang, Lin Li, Huawei Cai

**Affiliations:** 1 Department of Nuclear Medicine & Laboratary of Clinical Nuclear Medicine, West China Hospital, Sichuan University, Chengdu, 610041 P.R. China; 2 Department of Biochemistry & Molecular Biology, West China School of Basic Sciences & Forensic Medicine, Sichuan University, Chengdu, 610041 China; 3 College of Polymer Science and Engineering, State Key Laboratory of Polymer Materials Engineering, Sichuan University, Chengdu, 610065 P.R. China; 4 School of New Energy and Materials, Southwest Petroleum University, Chengdu, 610500 P.R. China; 5 Institute of Nuclear Physics and Chemistry, China Academy of Engineering Physics, Mianyang, 621900 P.R. China

**Keywords:** Hepatocellular carcinoma, Interventional radioembolization, Polydopamine, Lutetium-177, SPECT/CT

## Abstract

**Background:**

Transarterial radioembolization (TARE) with ^90^Y-labeled glass and resin microspheres is one of the primary treatment strategies for advanced-stage primary and metastatic hepatocellular carcinoma (HCC). However, difficulties of real-time monitoring post administration and embolic hypoxia influence treatment prognosis. In this study, we developed a new biodegradable polymer microsphere that can simultaneously load ^177^Lu and MgO nanoparticle, and evaluated the TARE therapeutic efficacy and biosafety of ^177^Lu-PDA-CS-MgO microspheres for HCC treatment.

**Methods:**

Chitosan microspheres were synthesized through emulsification crosslink reaction and then conducted surface modification with polydopamine (PDA). The ^177^Lu and nano MgO were conjugated to microspheres using active chemical groups of PDA. The characteristics of radionuclide loading efficiency, biodegradability, blood compatibility, and anti-tumor effectwere evaluated both in vitro and in vivo. SPECT/CT imaging was performed to monitor bio-distribution and bio-stability of ^177^Lu-PDA-CS-MgO after TARE treatment. The survival duration of each rat was monitored. HE analysis, TUNEL analysis, immunohistochemical analysis, and western blot analysis were conducted to explore the anti-tumor effect and mechanism of composited microspheres. Body weight, liver function, blood routine examination were monitored at different time points to evaluate the bio-safety of microspheres.

**Results:**

The composite ^177^Lu-PDA-CS-MgO microsphere indicated satisfactory degradability, biocompatibility, radionuclide loading efficiency and radiochemical stability in vitro. Cellular evaluation showed that ^177^Lu-PDA-CS-MgO had significant anti-tumor effect and blocked tumor cell cycles in S phase. Surgical TARE treatment with ^177^Lu-PDA-CS-MgO significantly prolonged the medial survival time from 49 d to 105 d, and effectively inhibited primary tumor growth and small metastases spreading. Moreover, these microspheres indicated ideal in vivo stability and allowed real-time SPECT/CT monitoring for up to 8 weeks. Immunostaining and immunoblotting results also confirmed that ^177^Lu-PDA-CS-MgO had potential in suppressing tumor invasion and angiogenesis, and improved embolic hypoxia in HCC tissues. Further evaluations of body weight, blood test, and pathological analysis indicated good biosafety of ^177^Lu-PDA-CS-MgO microspheres in vivo.

**Conclusion:**

Our study demonstrated that ^177^Lu-PDA-CS-MgO microsphere hold great potential as interventional brachytherapy candidate for HCC therapy.

**Graphical Abstract:**

Polymer composite microspheres loading ^177^Lu radionuclide and MgO nanoparticles for interventional radioembolization therapy and real-time SPECT imaging of hepatic cancer.

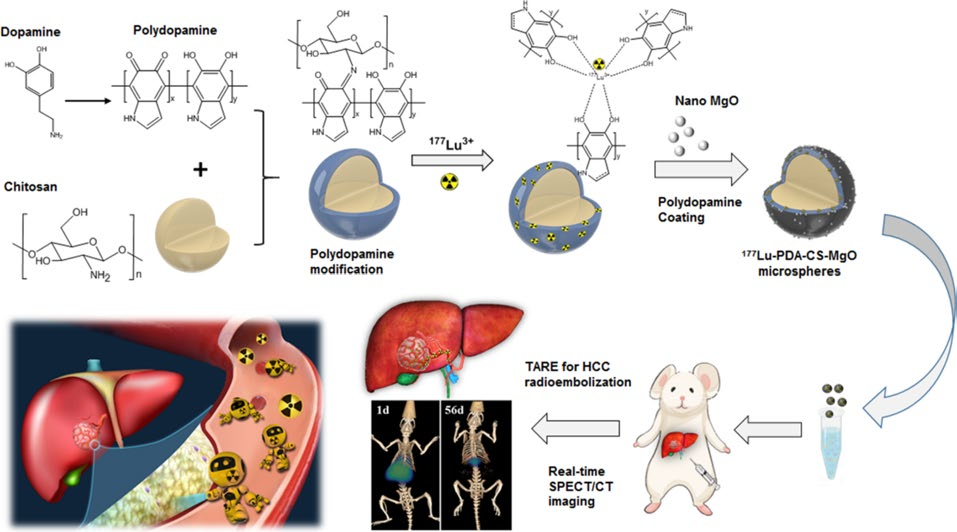

**Supplementary Information:**

The online version contains supplementary material available at 10.1186/s40824-023-00455-x.

## Introduction

Hepatocellular carcinoma (HCC) is a serious malignant carcinoma with extremly high morbidity and mortality in worldwide [1]. Interventional brachytherapy with transarterial chemoembolization (TACE) and transarterial radioembolization (TARE) via the main tumor arteries enables administration of high concentration of cytotoxic agents within tumor lesion and minimal systemic side effects, and has become one of the primary treatment strategies for advanced-stage HCC [2, 3]. Compared to TACE, TARE therapy with radionuclide has been reported to improve disease progression and extend overall survival of patients [4, 5].

Food and Drug Administration (FDA) has approved ^90^Y-labeled glass and resin microspheres for treatment of primary and metastatic hepatic cancer. These microspheres offer impactful radiation effect on tumor lesion by high pure β ray energy (T_1/2_=2.23 d, average E_β−_= 0.937 MeV) [6]. However, there are also some drawbacks, such as improper occlusion in capillaries, difficulties of real-time monitoring post administration in vivo [7], and radiotherapy resistance caused by thrombotic hypoxia [8]. Thus, the investigation of alternative biodegradable microspheres combined with therapeutic radionuclides attracts extensive attention. Various microspheres loaded with ^111^In [9], ^131^I [10–12], ^166^Ho [13], or ^177^Lu [14] were designed and evaluated in recent years. Our previous research also indicated ^131^I labeled chitosan-collagen composite microsphere could effectively inhibit tumor growth through TARE treatment and support in vivo single photon emission computed tomography (SPECT) imaging post surgery [12].

Similar to iodine-131(T_1/2_=8.4 d, average E_β−_=0.608 MeV), lutetium-177 (^177^Lu, T_1/2_=6.67 d, average E_β−_=0.498 MeV) has garnered extraordinary attention as “theranostic” radionuclide due to its unique properties of therapeutic β-ray energy and lower γ-ray energy of 113 KeV and 208 KeV for SPECT visulization better than ^131^I [15]. In recent years, ^177^Lu radiopharmaceuticals have achieved outstanding anti-caner effect in radioligand therapy area of neuroendocrine and prostate cancer in clinic, and thus, the potential applications in more therapeutic areas are worth anticipated [16, 17]. The chelation strategy is crucial for radiolabeling of ^177^Lu. Polyazo heterocyclic compounds, such as 1,4,7,10-Tetraazacyclododecane-1,4,7,10-tetraacetic acid (DOTA) and 1,4,7-triazacyclononane-N,N’,N’’-triaceticacid (NOTA), are common agents utilized in the preparation of ^177^Lu radiopharmaceuticals [15]. However, recently, we noticed another multifunctional biomaterial, also indicated metal chelating capability and potential utilization in microsphere preparation. The polydopamine (PDA), which is the product of natural melanin analog dopamine through autooxidation [18]. PDA is an ideal versatile tool for surface modification in a variety of biomaterials to give them unique characteristics to load chemotherapeutic drugs or metal ions including Cu^2+^ and Fe^3+^, which attracts our interest to develop its potential in ^177^Lu radiolabeling [19]. Embolization therapy always results in local hypoxia of tumor, which leads to adaptive changes of radiotherapy resistence and tumor recurrence [8]. In view of this, relieve hypoxia after TACE or TARE treatment is one of the keys to improve the efficacy of vascular embolization therapy for HCC. Recently, alkaline degradation products produced by biodegradable magnesium alloys or magnesium oxide (MgO) nanoparticles have been reported to effectively neutralize acidic tumor microenvironment, and improve hypoxia in tumor region by inhibiting hypoxia inducible factor-1α (HIF-1α) pathway [20, 21].

**Fig. 1:**
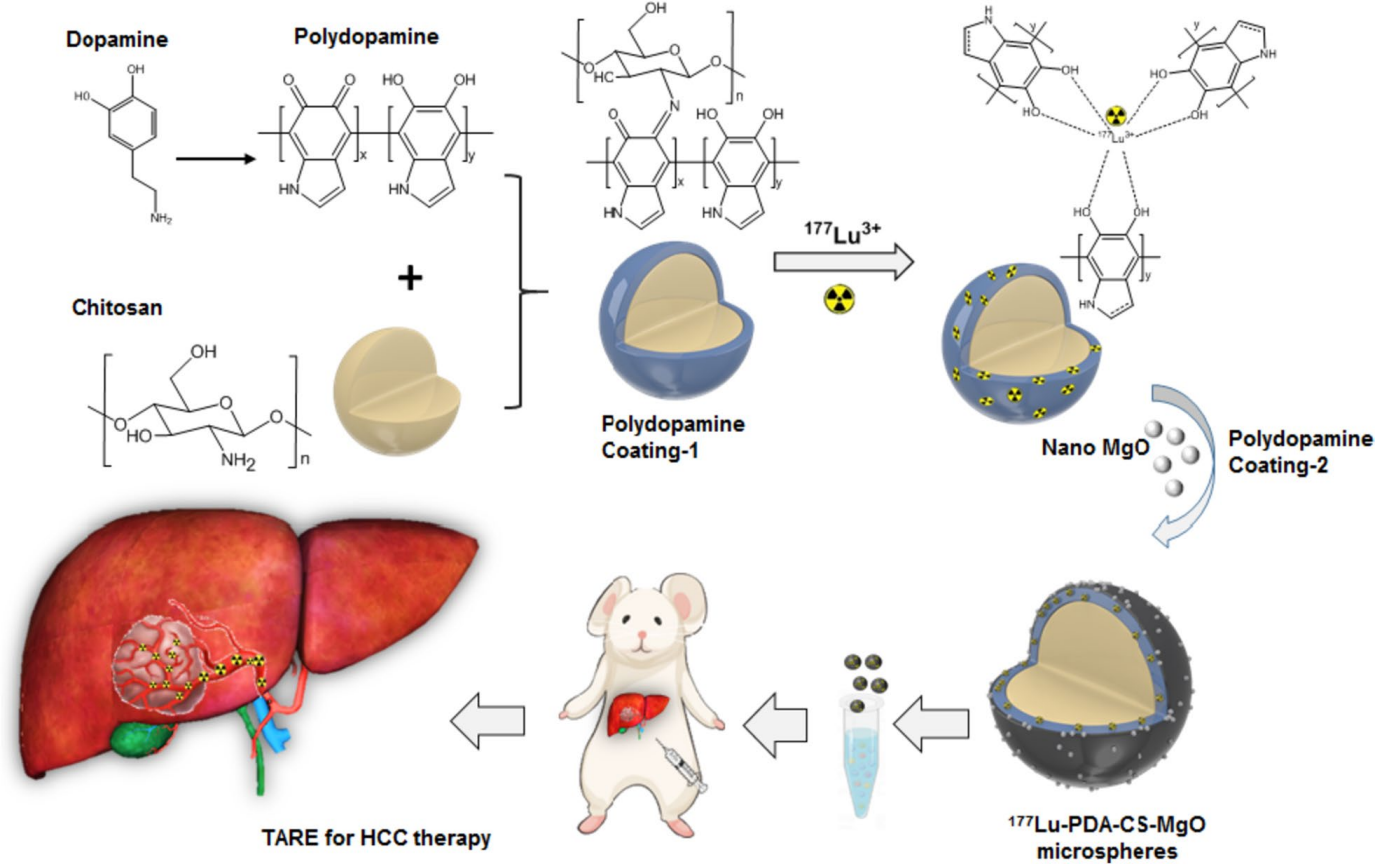
Schematic abstract of study. Polydopamine (PDA) was formed by self-polymerization of dopamine and coated on the surface of chitosan microspheres (CS). Then, ^177^Lu was chelated to PDA-CS microspheres by catechol groups and nano-MgO particles were modified on the surface to prepare ^177^Lu-PDA-CS-MgO microspheres. The radioactive microspheres were injected into rats through hepatic artery for embolization of primary HCC

Based on these advantages of ^177^Lu in brachytherapy and possible hypoxia alleviation of MgO, in this study, a novel versatile polydopamine-chitosan composite microsphere was prepared, for loading ^177^Lu and the nano-MgO particles through PDA surface modification, and the interventional therapeutic effect for HCC was evaluated (Fig. 1).

## Materials and methods

### Materials

Chitosan (CS) (50–190 KDa, deacetylation greater than 50%), dopamine (DA), liquid paraffin, sorbitan monooleate (Span-80) and N-nitrosodiethylamine (DEN) were supplied by Sigma-Aldrich (St Louis, MO, USA). No-carrier-added (n.c.a.) ^177^LuCl_3_ was produced via indirect ^176^Yb(n,γ)^177^Yb→^177^Lu with thermal neutron of reactor by the Institute of Nuclear Physics and Chemistry (INPC, China), China Academy of Engineering Physics [22, 23]. Human umbilical vein endothelial cell line (HuVEC) and hepatic cancer cell line HepG2 were purchased from Chinese Academy of Sciences Cell Bank (Shanghai). Cells were cultured by RPMI-1640 medium with 10% fetal bovine serum (FBS) and 1% penicillin-streptomycin in 5% CO_2_ atmosphere at 37 °C.

### Preparation of microspheres

The core CS microspheres (CSs) were synthesized using an emulsification crosslink reaction following our previous study [12]. About 80 mg of chitosan was dissolved in a 10 mL of 3% acetic acid solution, and slowly added into 20 mL of liquid paraffin containing 4.2% (v/v) Span-80. The mixture was stirred at 500 rpm for 1 h at room temperature (RT) to form the w/o emulsion. Subsequently, 500 µL of glutaraldehyde was added dropwise and stirred continuously for another 2 h to catalyze crosslinking and solidification of microspheres. The oil phase was removed from CSs by isopropanol washing. The product mixture was filtered through a 100-µm mesh to eliminate unformed large particles and freeze-dried for further usage. For the first PDA coating, 5 mg of CSs were dissolved in 5 mL PBS (20 mM), and then added 10 mg of dopamine (10 mg/mL) and 20 mM Tris-HCl solution to adjust pH value to reach 8.5; PDA-CS microspheres would be obtained under stirring for 2 h. For radiolabeling of ^177^Lu, 10 mg of PDA-CS was dissolved into 10 mL PBS, and added with 10 mg dopamine and 0.5 mL of ^177^LuCl_3_ (370–480 MBq in 50 mM HCl) for 4 h. The microspheres were washed several times with PBS to remove unbound radionuclides and separated through centrifugation at 6000 rpm for 5 min. Nano-MgO particles (diamter of -10 nm) were prepared by the liquid precipitation method. To facilitate the ^177^Lu-PDA-CS-MgO, 5 mg of MgO nanoparticles, 10 mg of dopamine and 100 mg of ^177^Lu-PDA-CS were added into 10 mL of Tris-HCl solution (10 mM, pH = 8.5) under stirring for 2 h. The Non-radioactive ^175^Lu was used to replace the ^177^LuCl_3_ solution and prepare Lu-PDA-CS-MgO for characteristic evaluation.

### Characteristics of microspheres

Particle size was assessed by Mastersizer 3000E Laser particle size analyzer (Malvern Panalytical ltd, Kassel, Germany), and biodegradability of microspheres were examined using JSM-390LV scanning electron microscopy (SEM) (JEOL, Tokyo, Japan). For number counting of microspheres, 1 mg of microspheres were diluted in ddH_2_O and the calculating assessment was completed by a Countstar Biotech device (ALIT Biotech, Shanghai) (*n* = 5). Fourier-transform infrared (FTIR) spectrometry, zeta potential and Thermogravimetric Analysis (TGA) were performed on PDA, CS, and PDA-CS to examine the newly formed chemical conjugates. Energy dispersive spectroscopy (EDS), element mapping scanning, and X-ray photoelectron spectroscopy (XPS) were carried out to quantify the contents of C, O, N, Mg and Lu in microspheres.

To assess in vitro stability, 18.5 MBq of ^177^Lu-PDA-CS-MgO microspheres suspension was mixed with 1 mL of FBS and PBS respectively at 37 °C for up to 336 h. Periodical centrifugation was performed, and the radioactivities of supernatant and sedimentation were measured to evaluate the not complexed ^177^Lu. To assess in vivo stability, a dose of 7.4 MBq of microspheres were transarterally injected into normal rats. The percentage of injected dose per gram of tissue (%ID/g) in major organs were calculated at 1, 3, and 7 d post-surgery (*n* = 3 for each group), including blood, heart, liver, spleen, lung, renal, stomach, intestine, muscle, and bone.

### Biosafety assessment of microspheres in vitro

Hemolysis and coagulation test was conducted to evaluate blood compatibility of non-active PDA-CS-MgO and PDA-CS microspheres following previous literature [11]. For hemolysis evaluation, 2 mL of rat whole blood with heparin sodium was mixed with an equal volume of saline and centrifuged at 1500 rpm for 10 min to collect blood red cells suspension (RCS). 0.2 mL of RCS diluent (2.0% in saline) was added to saline dispersion PDA-CS-MgO and PDA-CS (1, 5, and 10 mg/mL). 0.9% saline was used as negative control, and the ultrapure water was considered as positive control. The mixtures were centrifuged at 2000 rpm for 10 min after 1 h culturing. The supernatant was used for the determination of optical density (OD value) by a microplate reader at the wavelength of 540 nm [24]. The hemolysis Ratio (HR) was calculated using the following equation: *HR= (ODe-ODn)/(ODp-ODn)*. ODe, ODp and ODn were the OD values of experiment group, positive control and negative control, respectively.

In the coagulation experiment [11], RCS diluent was added to PDA-CS-MgO and PDA-CS saline dispersion, followed by the addition of 20 µL CaCl_2_ solution (0.2 M) to initiate clotting. The beaker was further added 1 mL of distilled water and incubated at 37 °C for 10 min. RCS diluent with distilled water and was set as control group. Finally, the OD540 of the solution was measured. The blood coagulation index (BCI) can be calculated by the following equation: *BCI = ODe/ODc*. ODe and ODc were the OD values of experiment group and control group, respectively.

The cytotoxic effect of microspheres on normal cells was evaluated in HuVEC cells. 1 × 10^5^ cells/well were seeded in 6 well plates and 1, 3, 5, and 10 mg of nonradioactive PDA-CS-MgO and PDA-CS were added to wells and incubated for 24 h. Each concentration was performed in triplicates. Then, cell viabilities were measured using Cell Counting Kit-8 (CCK-8) assay.

### In vitro anti-cancer evaluation

The anti-cancer effect of microspheres were evaluated by live/dead cell staining and flowcytometry. HepG2 cells was seeded in 6 well plates with density of 1 × 10^5^ cells/well for 24 h for attachment. Then, 1 mg of PDA-CS, PDA-CS-MgO, ^177^Lu-PDA-CS (0.74, 1.85, 3.7 MBq), ^177^Lu-PDA-CS-MgO (0.74, 1.85, 3.7 MBq) microspheres were added into wells, respectively. After 24 h incubation, live/dead cell staining were applied with Calcein AM-PI staining (KeyGEN BioTECH, Jiangsu, China) and captured by flourescent microscope. Brifely, cells were washed by PBS, combined with buffer solution and incubated for 30 min with 1 mL Calcein AM-PI to evaluate the percentage of dead cells. The live cells were stained green while dead cells appeared red. Apoptosis analysis was performed using Annexin V-APC staining (KeyGEN) after treatment. Cells were washed by cold PBS, combined with buffer solution and incubated for 15 min with Annexin V-APC to determine the percentage of apoptotic cells. Flow cytometry data were analyzed with FlowJo VX software (Tree Star Inc., Ashland, OR, USA). Cell cycle progression was analyzed by PI/RNase staining (KeyGEN). The treated cells were immobilized at 4 °C overnight with 75% ethanol, washed thoroughly with PBS, and incubated with PI/RNase staining solution for 30 min. Then, the DNA ratios of different stages were calculated by ModFit LT™ (Verity Software House, Topsham, ME).

### Animal modeling and interventional radioembolization

All the animal experiments were performed in compliance with protocol approved by the Animal Care and Use Committees guidelines in West China Hospital and Sichuan University (2016-028A). SD rats (150–200 g, 6–8 w) were sourced from Chengdu Dossy Experimental Animals Co., Ltd and used as experimental subject. The primary HCC models were established by 0.01% nitrosodiethylamine chemical induction through oral administration for 8–10 w. Tumor growth was monitored by MRI (Signa 3.0 T, GE Health, USA) according to our previous method [12]. Rats with no more than 3 intrahepatic nodules in all slices were selected for further study. TARE was performed through transarterial administration, and the rats were divided into four groups (*n* = 5 for each group): ^177^Lu-PDA-CS-MgO group (37 MBq), ^177^Lu-PDA-CS group (37 MBq), PDA-CS group and sham operation control group with injection of sterilized saline. Additionally, another group was injected with 37 MBq of ^177^Lu-Lipiodol as another control to evaluate the biodistribution of dissociative ^177^Lu under this administration (*n* = 3). The dose administration was calculated according to our previous study [12]. The absorbed dose can be calculated by the following equation:

$$\boldsymbol A\boldsymbol b\boldsymbol s\boldsymbol o\boldsymbol r\boldsymbol b\boldsymbol e\boldsymbol d\boldsymbol\;\boldsymbol d\boldsymbol o\boldsymbol s{\boldsymbol e}_{\mathrm{liver}}=\frac{\boldsymbol I\boldsymbol n\boldsymbol j\boldsymbol e\boldsymbol c\boldsymbol t\boldsymbol e\boldsymbol d\boldsymbol\;\boldsymbol d\boldsymbol o\boldsymbol s\boldsymbol e(\boldsymbol G\boldsymbol B\boldsymbol q)\times\;\boldsymbol S\;\boldsymbol v\boldsymbol a\boldsymbol l\boldsymbol u\boldsymbol e(\boldsymbol G\boldsymbol y/\boldsymbol G\boldsymbol B\boldsymbol q)}{\boldsymbol l\boldsymbol i\boldsymbol v\boldsymbol e\boldsymbol r\boldsymbol\;\boldsymbol w\boldsymbol e\boldsymbol i\boldsymbol g\boldsymbol h\boldsymbol t\;(\boldsymbol g)}$$

### In vivo anti-HCC evaluation

The tumor inhibition efficiency was monitored by MRI imaging post administration for up to 5 w. Whole body SPECT/CT imaging was performed using GE Discovery NM670 (GE Health, USA) to investigate the biodistribution and stability of ^177^Lu-PDA-CS-MgO microspheres in vivo. Helical SPECT images were obtained using a double-headed camera with MPEG collimator (energy peak 208 keV ± 10%) in 30 projections over 15 min. CT images were acquired in 30 projections with a 1000-ms exposure time using a 45-kV X-ray source over 5 min. Radioactive distribution of the whole body was reconstructed using a two-dimensional, iterative, ordered-subset expectation maximization algorithm, and these images were fused with CT images using Xeleris 4.0 software (GE Health).

The median overall survival time was calculated until the animals reached the maximum allowable tumor burden and natural death. Body weight, liver function, and blood indices were recorded during this period. Then, liver tissues were harvested for immunohistochemical analysis. Consecutive 5-µm thickness slices were prepared to stain with hematoxylin & eosin (H&E), TUNEL, Ki-67, CD31, and HIF-1α for pathological evaluation, respectively. Western blot analysis of HIF-1α, VEGFRα, Bcl-2 and Bax were conducted on harvested tumor tissues of each group. Semi-quantitative analysis of these immunohistochemical staining was conducted by Image J software. H&E staining of other major organs (heart, lung, spleen, kidney, stomach and intestine) were monitored to investigate the side effect.

### Statistical analysis

All of the data were expressed as the mean ± standard deviation (SD) unless specified otherwise. Student’s t test or ANOVA was performed for statistical analysis and Kaplan–Meier was used for calculation of survival analysis. Differences at the 95% confidence level (*p* < 0.05) were considered significant. The statistical analysis was performed using SPSS 22.0.

## Results

### Microspheres characterization

**Fig. 2:**
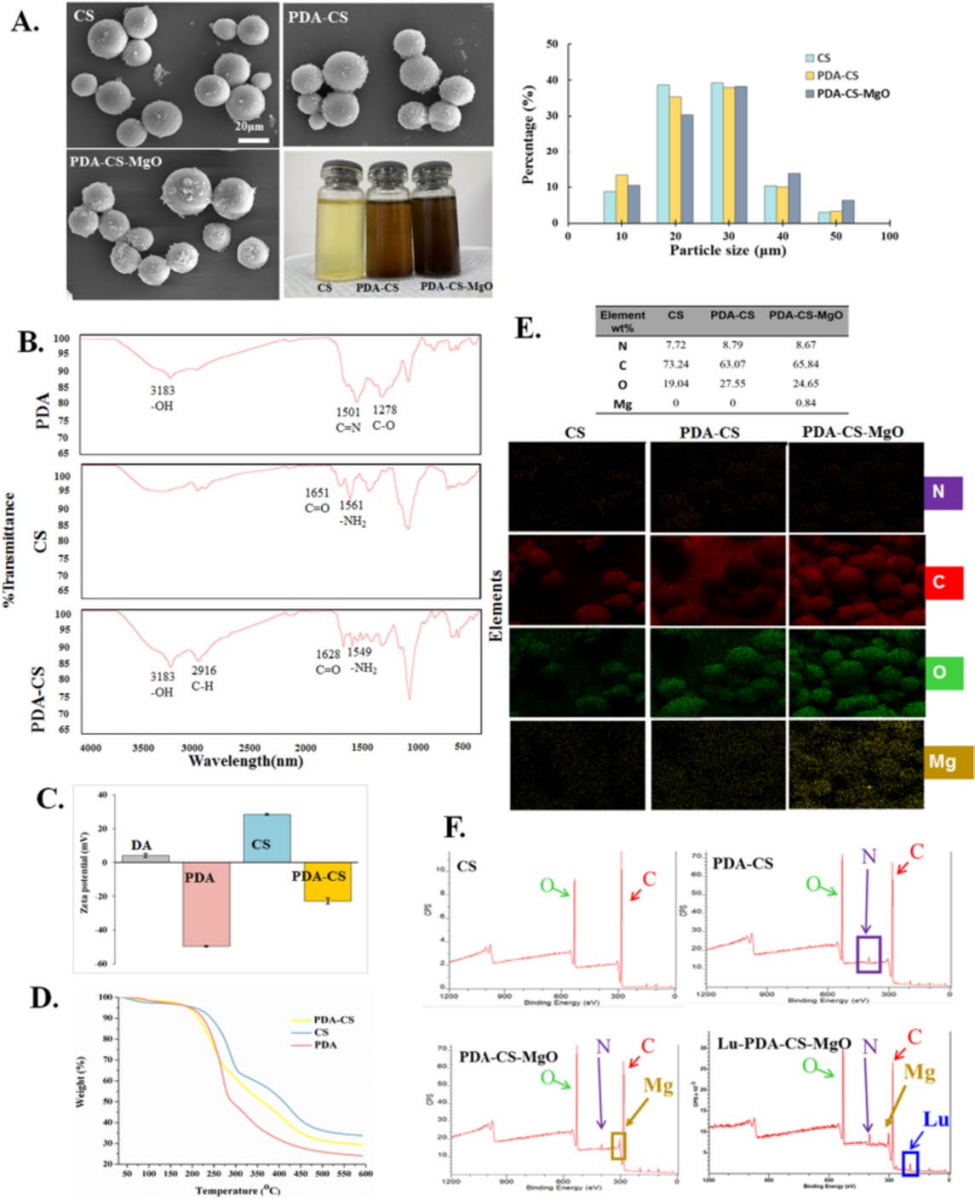
Characteristics of microspheres. **A.** SEM images and particle size assessment of CS, PDA-CS and PDA-CS-MgO. **B.** FTIR spectral of CS, PDA, and PDA-CS. **C.** Zeta potential of DA, CS, PDA, and PDA-CS. **D.** TGA analysis of weight loss curves under different temperatures for CS, PDA and PDA-CS. **E.** Element mapping of N, C, O and Mg elemetns in microspheres. **F.** XPS wide spectral of O-1s, C-1s, N-1s, Mg KLL and Lu-4d in microspheres

In this study, CS, PDA-CS and PDA-CS-MgO microspheres were prepared, and the radiolabeling feasibility was evaluated. As illustrated in Fig. 2A, these microspheres showed smooth spherical shape and desirable diameter of 20–30 μm, and the calculating assessment indicated approximate 3 × 10^5^ particles in every milligram of microspheres. Settlement experiment indicated ideal suspending ability in saline, making them ideal for injection purposes. FTIR indicated that distinct absorption peaks at 1200–1700 cm^−1^ and 2900–3200 cm^−1^ in PDA-CS, which suggested newly formed chemical bonds between CS and PDA through a Schiff base reaction (Fig. 2B). The major absorption peaks were observed in the wavelength from 4000 to 400 cm^−1^. PDA peaks are located at 1278 cm^−1^, 1501 cm^−1^ and 3183 cm^−1^, which are correspond to C-O, C = N or/and C = C and -OH or/and N-H vibrational modes, respectively [25, 26]. The absorption peak of CS at 1651 cm^−1^ is characteristic peak of carbonyl (C = O-NHR) and the peak of 1561 cm^−1^ is amine (NH_2_) [27]. Compared to CS FTIR spectra, the formation of PDA coating around CS leads to a decreased peak at 1549 cm^−1^ in the spectrum of PDA-CS, which may indicate consuming of -NH_2_ groups in CS due to Schiff base reaction. Moreover, more new absorption peaks are observed from 1275 cm^−1^ to 1500 cm^−1^ compared to pure CS. The absorption peak at 2916 cm^-1^ belongs to the peak of the C-H bond stretching vibration [27]. Zeta potential of PDA was negative charged as -49.7 ± 0.4 mV, which was induced by the oxidative self polymerization of DA (4.06 ± 1.21 mV). However, PDA-CS indicated an intermediate zeta potential of -22.8 ± 1.65 mV between PDA and CS (38.37 ± 0.51 mV), which may be due to the ingredients neutralization and is similar to previous report of PDA conjugates [28] (Fig. 2C). TGA result also indicated distinctive thermal decomposition curve of PDA-CS compared to CS and PDA, which can be explained by the fact that the weight loss of CS was mainly attributed to the complete decomposition of the chitosan molecules, while for PDA-CS, it was ascribed to the breakdown of chitosan and the decomposition of PDA [29] (Fig. 2D). EDS mapping revealed presence and varying amounts of C, N, O, and Mg in different microspheres (Fig. 2E). XPS analysis was employed to study the constituent elements of C, O, N, Mg and Lu in different samples (Fig. 2F). Non-radioactive ^175^Lu-PDA-CS-MgO was prepared for XPS spectral. C-1s shows peak at 284.5 eV [30], O-1s shows peak at 531.7 eV [30], and N-1s shows peaks at 396.2 and 389.7 eV [31]. Mg KLL peak was detected at 305.4 eV [32]. Lu-4d shows peaks at 206.5 eV and 194.8 eV, and confirmed the presence of Lu in trivalent oxidation state [30].

### In vitro biosafety, stability and anti-HCC effect of microspheres

**Fig. 3:**
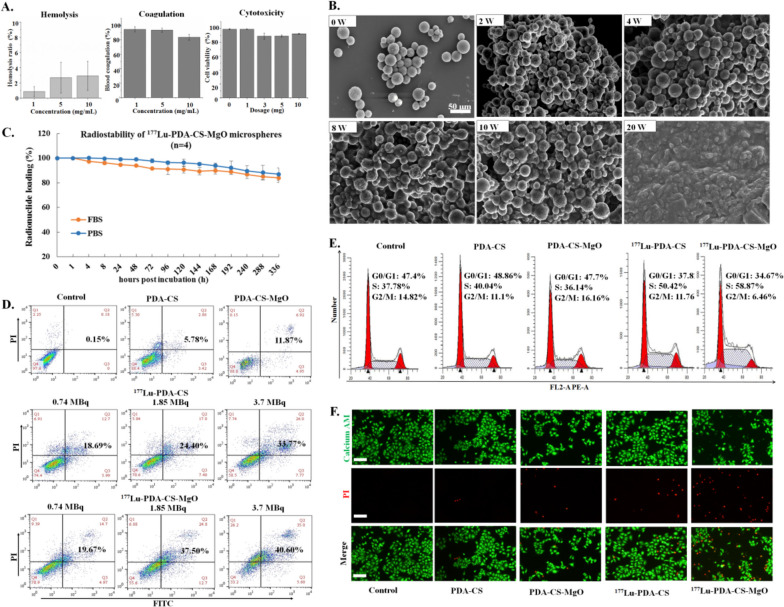
In vitro evaluation of biosafety, stability and anti-HCC effect of different microspheres. **A.** Hemolysis rate, blood coagulation index and cellular cytotoxicity test for PDA-CS-MgO. **B.** SEM image of PDA-CS-MgO in rat serum at 2, 4, 8, 10, and 20 w. **C.** Radiochemical stability evaluation of ^177^Lu-PDA-CS-MgO. **D.** HepG2 cell apoptosis analysis for different microspheres. **E.** HepG2 cell cycle analysis after microspheres treatment. **F.** Live/dead cell staining. Live cells were stained as green while dead cells appeared red, and the scale bar was 100 μm

As shown in Fig. 3A, hemolysis rate of PDA-CS-MgO was found to be lower than 5% under 10 mg/mL treatment, indicating favorable blood compatibility. Similarly, blood coagulation index were more than 80%, which would be suitable for embolization and avoid unexpected thrombus in surgery. Then, HuVEC viability was above 90% when exposed to varying PDA-CS-MgO doses, suggesting favorable biological safety as PDA-CS (Supplement Fig. 1A&B&C). Then, as shown in Fig. 3B, the composite PDA-CS-MgO microspheres began to polymerize in serum since 2 w. Subsequently, boundaries of microspheres became misty and interweave to each other after 8 w, and showed remarkable degradation after 20 w; which was similar to the biodegradative cycle of PDA-CS (Supplement 1D). The radiolabeling efficiency of ^177^Lu to PDA-CS-MgO microspheres was 87.10 ± 5.52% and the maximum loading dose of radionuclide was 45.51 ± 1.85 MBq/mg (*n* = 10). ^177^Lu-PDA-CS-MgO remained radiochemical purity of 85.15 ± 0.91% in FBS and 88.65 ± 0.20% in PBS after 336 h, indicating satisfactory stability in vitro (Fig. 3C).

The pro-apoptotic effect of microspheres PDA-CS, PDA-CS-MgO, ^177^Lu-PDA-CS, and ^177^Lu-PDA-CS-MgO to hepatic cancer HepG2 cells were assessed through FITC-Annexin V/PI flowcytometry. As shown in Fig. 3D, no significant apoptotic effect was investigated in the cells after 24 h incubation with non-radioactive PDA-CS microspheres (5.78%), while the addition of MgO slightly enhanced the pro-apoptotic capability up to 11.87% for PDA-CS-MgO, which is probably caused by the alkaline pH value from the degradation products of MgO. However, radioactive microspheres indicated dose-dependently apoptosis to HepG2 cells, with the highest apoptotic rate of 40.60% under treatment by 3.7 MBq of ^177^Lu-PDA-CS-MgO. Further cell cycle analysis revealed that ^177^Lu radiation effect inhibited HepG2 proliferation at S phase, while the non-radioactive microspheres did not influence the cell cycle (Fig. 3E). Calcium AM/PI staining results were consistent with the flowcytometry results, and ^177^Lu-PDA-CS-MgO induced the most significant cytotoxicity to HepG2 cells in comparison to other groups (Fig. 3F). All these findings indicate that ^177^Lu-PDA-CS-MgO holds effective anti-cancer ability to eradicate HCC cells in vitro.

### In vivo biodistribution and SPECT/CT imaging of ^177^Lu-PDA-CS-MgO microspheres

**Fig. 4:**
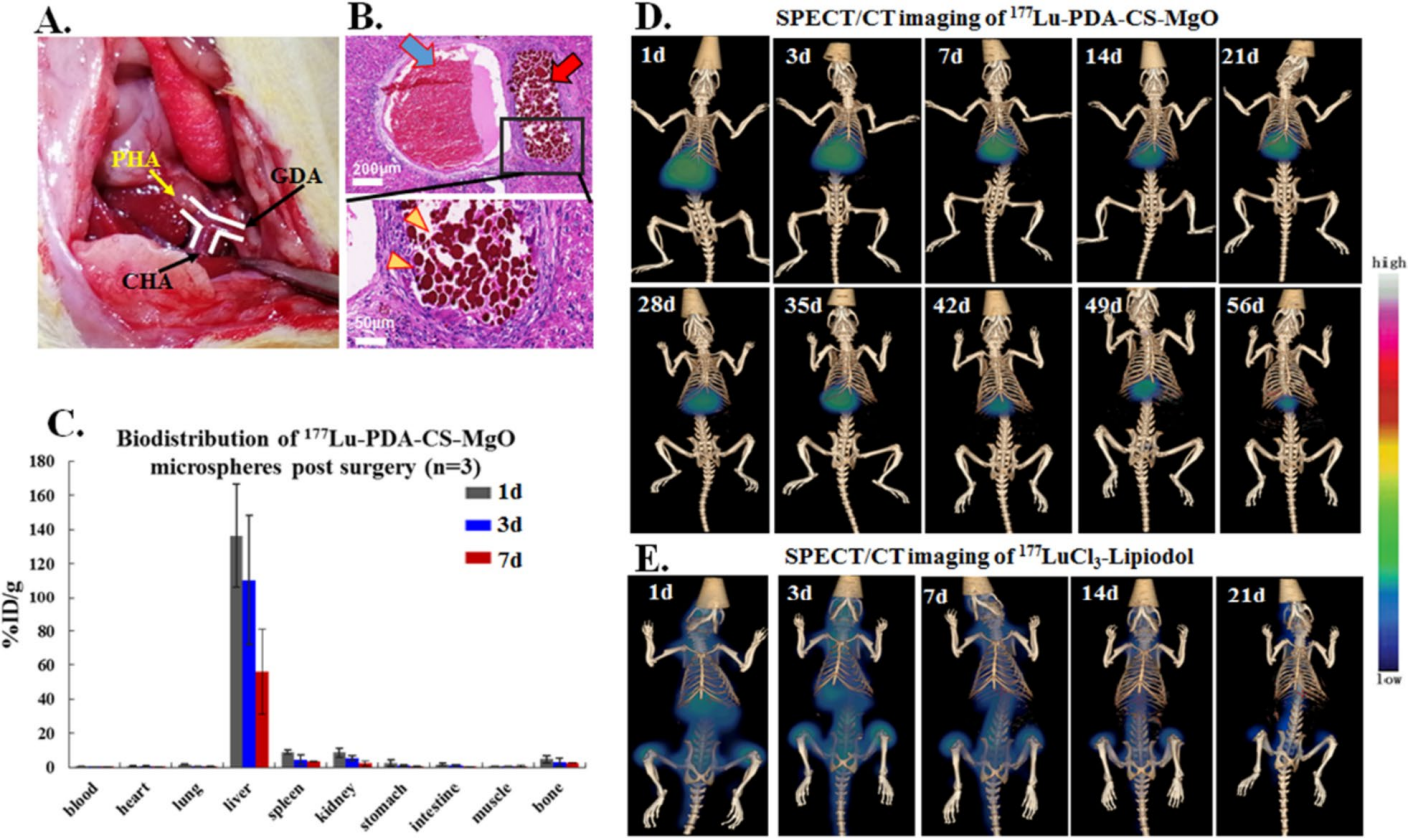
TARE administration and in vivo biodistribution of ^177^Lu-PDA-CS-MgO microspheres in rat models. **A.** TARE administration via PHA injection. PHA, proper hepatic artery. CHA, common hepatic artery. GDA, gastroduodenal artery. **B.** H&E staining indicated successful hepatic arteral embolization by microspheres. Blue arrow points out the hepatic vein, red arrow points out the artery, and yellow triangles point out the embolized microspheres in arterial. **C.** In vivo biodistribution of ^177^Lu-PDA-CS-MgO in major organs of rat models at 1, 3, and 7 d post administration (*n* = 3). **D.** Whole-body SPECT/CT imaging of ^177^Lu-PDA-CS-MgO in vivo. **E.** SPECT/CT imaging of ^177^LuCl_3_-Lipiodol after PHA administration

Interventional TARE was performed by a laparotomy following the previous method [12], and 3–5 × 10^5^ particles of ^177^Lu-PDA-CS-MgO with 37 MBq radioactivity was administrated into rat models through proper hepatic artery (PHA) (Fig. 4A).

After 5 w, H&E staining of postoperative liver tissues revealed stacked microspheres in hepatic arterial only, suggesting successful transarterial injection and embolization (Fig. 4B). In vivo biodistribution demonstrated that the radioactivity of ^177^Lu-PDA-CS-MgO predominantly accumulated in the liver without apparent uptake in the other tissues post administration. The slight radioactivity in spleen is probably caused by the trace amount of microspheres in regurgitative blood after administration. Then, decomposed ^177^Lu-PDA induced slight radioactivity levels in spleen and kidney during the process of spleen phagocytosis and renal excretion, while free ^177^Lu ions resulted in the slight radioactivity in bones (Fig. 4C). After TARE treatment, SPECT/CT scanning was conducted to monitor distribution of microspheres in vivo. The imaging results (Fig. 4D) revealed that ^177^Lu-PDA-CS-MgOs were perfectly accumulated in the liver for up to 56 d post-injection, which was consistent to biodistribution result. We also investigated the biodistribution of unchelated ^177^Lu from ^177^LuCl_3_-Lipiodol mixture in vivo. Nevertheless, the radioactivity of ^177^Lu spread from liver to bone joints in less than 24 h post-injection and eliminated after 7 d, suggesting the unstable localization of radioisotopes without the help of composite microspheres. Moreover, white blood cell (WBC) and platelet (PLT) in ^177^Lu-Lipiodol group displayed dramatical suppression in 3 d post treatment and then recovered after 2 w, which was related to myelosuppression induced by free ^177^Lu in whole body (Supplement Fig. 2) [33]. However, there were no significant differences in the other ^177^Lu-PDA-CS-MgO or ^177^Lu-PDA-CS groups compared to normal control group. All these results presented that ^177^Lu-PDA-CS-MgO exhibits satisfactory target organ biodistribution, stability, and biosafety in vivo.

### In vivo anti-cancer effect of ^177^Lu-PDA-CS-MgO microspheres

**Fig. 5:**
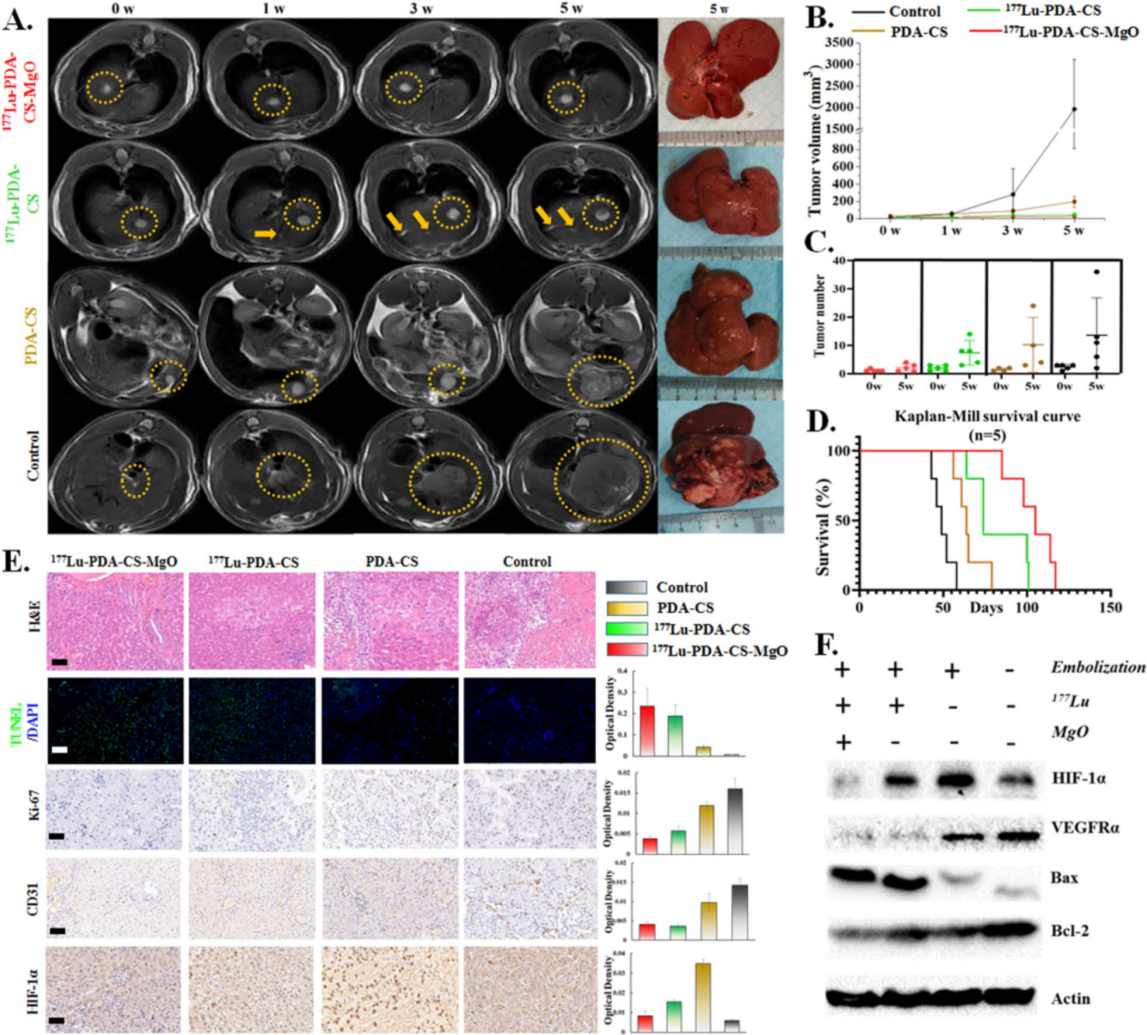
Evaluation of the in vivo anti-tumor efficacy of ^177^Lu-PDA-CS-MgO microspheres. **A.** Representative MRI monitoring of rat livers in 5 w post radioembolization. Yellow circles point out the primary focus and arrows point out new tumor lesions during treatment. **B.** Tumor volume in each group at 0 w, 1 w, 3 w and 5 w. **C.** Tumor numbers in each group at 0 w and 5 w. **D.** Survival curves of rats in each group. **E.** Representative immunohistochemical staining and semi-quantitative analysis of H&E, TUNEL, Ki-67, CD-31 and HIF-1α in tissue samples post-treatment. The scale bar is 100 μm. **F.** Western Blot analysis of typical biomarkers, including HIF-1α, VEGFRα, Bax and Bcl-2 in liver tissues

The tumor growth in rats was monitored by MRI and gross specimens after surgical radioembolization. Tumor volumes and numbers were assessed based on MR images. As shown in Fig. 5A and B, the primary tumor burden in sham operation control group rapidly grew up after 5 w, which was serious and lethal. Embolization without radioactive therapy moderately suppressed the primary tumor growth as 198.1 ± 64.17 mm^3^, however, irreversible liver cirrhosis and a variety of small lesions were generated. ^177^Lu-PDA-CS and ^177^Lu-PDA-CS-MgO microspheres represented satisfactory anti-tumoral effect to primary tumor lesion. Primary tumor volumes post TARE treatment were 28.94 ± 8.51 mm^3^ for ^177^Lu-PDA-CS-MgO and 46.87 ± 14.01 mm^3^ for ^177^Lu-PDA-CS (*P* = 0.037), respectively. The number of visible lesions in MRI images was in accordance with the trend of tumor growth, control group indicated average of 14 (3 to 37) lesions, PDA-CS embolization indicated 10 (3 to 24) lesions, and ^177^Lu-PDA-CS indicated 8 (3 to 15) lesion after 5 w, respectively. However, ^177^Lu-PDA-CS-MgO indicated the best inhibiting capability to small metastases with only 2 (1 to 4) lesions by MR monitoring (Fig. 5C). Kaplan-Mill survival analysis showed a median survival time (MS) of 105 d (95% CI: 89.9–120, *p* < 0.001) for ^177^Lu-PDA-CS-MgO group, which was remarkably prolonged than that of ^177^Lu-PDA-CS group (MS = 74 d), PDA-CS group (MS = 64 d), and control group (MS = 49 d) (Fig. 5D).

Liver tissues were collected at 5 w post treatment for pathological and immunohistochemial analysis (Fig. 5E). H&E staining exhibited integrated liver cell structures and regular blood vessels in ^177^Lu-PDA-CS-MgO group compared to the other groups. However, cells structures in control group were presented highly abnormal as nesty and patchy with stroma. Tumor tissues stained with TUNEL and Ki-67 indicated more significant apoptosis and less carcinoma proliferation in ^177^Lu-PDA-CS-MgO treated samples. Tumor associated angiogenesis is usually considered as foundation for metastases, while the examination of tumor-angiogenesis biomarker CD31 indicated it was dramatically suppressed by ^177^Lu radiation by both immunohistochemical analysis and western blot. HIF-1α, a representative biomarker of hypoxia, was found to be highly expressed after PDA-CS treatment but inhibited under ^177^Lu radiation and MgO therapy. Immunoblotting result in Fig. 5F confirmed the potential antitumor mechanism of microspheres. Results from both HIF-1α immunohistochemical analysis and WB indicated that non-radioactive vascular embolization treatment resulted in more hypoxia area in tumor tissues. Western blot also revealed ^177^Lu radiation successfully induced increase of pro-apoptotic protein Bax and suppression of anti-apoptotic protein bcl-2, which is consistent to in vitro results. These findings indicated satisfactory anti-tumor capability of ^177^Lu-PDA-CS-MgO microspheres for HCC therapy.

**Fig. 6:**
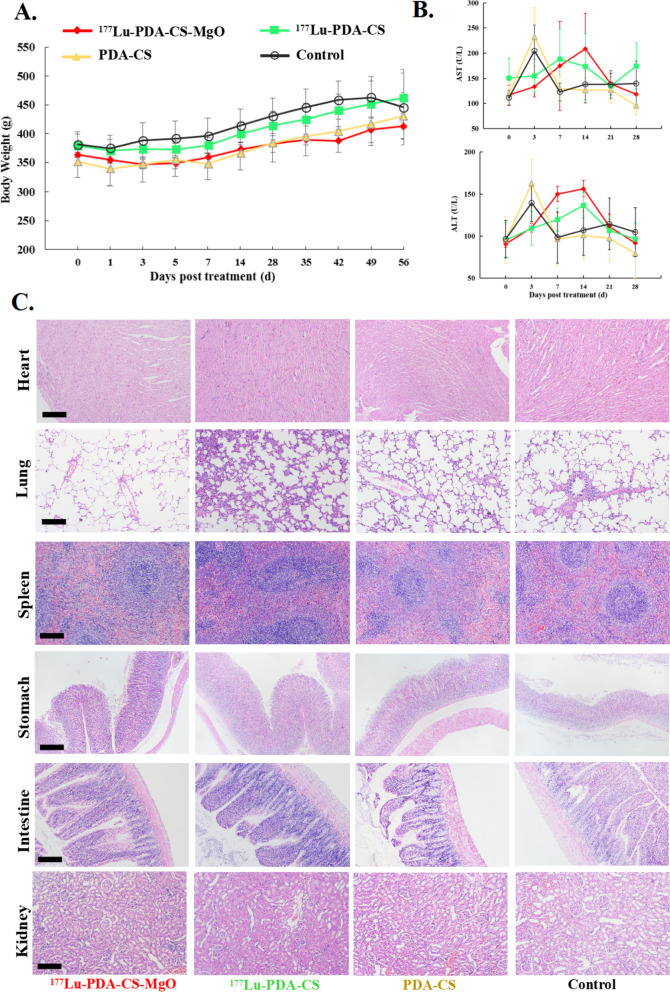
**A.** Body weight curves of rats in each group after surgery. **B.** ALT and AST changes in each group after surgery. **C.** HE staining of heart, lung, spleen, intestine, and kidney in each group. The scale bar is 200 μm

Furthermore, we also assessed the biosafety of microspheres in vivo by determing the body weights, liver function index, and physiological changes of major extrahepatic organs. The animal body weights fluctuated within normal ranges for each group (Fig. 6A). The liver function indicators AST and ALT showed no significant difference between the control group and any of the treatment groups (Fig. 6B). Then, no obvious pathological changes were observed in the heart, lung, spleen, intestine, or kidney in any of the groups, as demonstrated in Fig. 6C. All these results indicated satisfactory biosafety of ^177^Lu-PDA-CS-MgO microspheres for radioembolization in vivo.

## Discussion

Although TARE has become one of the primary treatment strategies for advanced-stage HCC, developing potential degradable biomedical materials, suitable therapeutic nuclides and inspired improvement of therapeutic efficacy are the focus of current field. In view of this, we developed a kind of new biodegradable composited microsphere that can simultaneously load therapeutica radionuclide ^177^Lu and hypoxia alleviator MgO nanoparticle. We also evaluated the TARE therapeutic efficacy, SPECT/CT imaging capability and biosafety of ^177^Lu-PDA-CS-MgO microspheres for HCC treatment.

Currently, a growing number of biocompatible materials have been developed for embolization studies [34]. Chitosan, a commonly used biomedical material, is known for its non-toxicity, good biocompatibility, and degradability [35]. A variety of studies confirmed that chitosan could be an ideal biomaterial as antiseptic dressing, drug carrier and embolization for medical applications [36, 37]. PDA is a reliable surface coating material, or can be synthesized as nano-platform for loading ^131^I, as it exhibits excellent both in vivo and in vitro stability [38, 39]. Notably, the abundant catechol groups on surface structures of PDA are suitable to act as chelating sites for metal radionuclides, such as ^64^Cu and ^99m^Tc, showing extraordinary labeling rates and in vivo stability [19, 40]. Based on the advantages of ^177^Lu in brachytherapy (therapeutic β-ray energy and lower γ-ray energy), we selected ^177^Lu as therapeutic nuclides for embolization treatment. PDA was used to chelate ^177^Lu with satisfactory biocompatibility and in vivo and vitro stability. Thus, a simpler labeling process has been developed, compared with conventional DOTA chelation.

On the other hand, embolic hypoxia influence embolization treatment prognosis. Previous studies have revealed that tumors with hypoxic tend to exhibit increased invasiveness and radioresistance [41, 42], which is probably to explain the small metastatic lesions in ^177^Lu-PDA-CS group. To solve this problem, we introduced the nano-MgO particle to the versatile microsphere platform, which would gradually degrade and release magnesium ions under the acidic microenvironment in tumor sites [21]. Previous study reported that the introduction of biodegradable Mg would successfully alleviate hypoxia via blocking HIF-1α/CAIX pathway [20]. Alkaline degradation products of Mg could further neutralize the acidic microenvironment at the site of cancer lesions, thereby inhibiting cancer cells [20]. Meanwhile, metal nanomaterials themselves can be used to inhibit the growth of HepG2 cells, by incuring a variety of events such as mitochondrial damage, lysosome impairment, endoplasmic reticulum stress, and signalling pathway alterations [43, 44]. Our result confirmed the enhancement of Mg in improving the ^177^Lu radiation anti-tumor effect and prolonging survival time, suggesting ^177^Lu-PDA-CS-MgO is a potential interventional radioembolization candidate for HCC therapy.

There are still some drawbacks in this study. Firstly, in clinic, TARE is performed with femoral arterial catheterization with digital subtraction angiography (DSA) intervention method. Unfortunately, we were not able to obtain the special DSA-catheterization device for rodents, and had to perform the TARE with an invasive procedure, which may affect the survival time post treatment. Then, the heavy gravimetric proportion of ^90^Y labeled TheraSphere and SIR-Spheres did not allow similar administration method. We regretfully did not make a raise of direct comparasion data between ^177^Lu-PDA-CS-MgO and ^90^Y-labeled microspheres. However, we still estimate the anti-tumor effect by radiation dosimetry evaluation.

The rat liver mass was 12–15 g, the S value (Activity Radiation exposure conversion coefficient) of ^177^Lu in liver was 11.2 by digimouse voxel phantom [45]. Consequently, the absorbed dose for 37 MBq of ^177^Lu was calculated as 32–41 Gy, which is comparable to the recommended dose from European Association of Nuclear Medicine (EANM) ^90^Y-microsphere therapy guideline.

## Conclusion

In this study, we successfully developed a novel versatile biodegradable microsphere loading ^177^Lu radionuclide by distinctive polydopamine chelation. With satisfactory biocompatibility and stability, this composited ^177^Lu-PDA-CS-MgO microsphere indicates ideal anti-cancer effect and allows real-time visible SPECT/CT monitoring post surgery, suggesting it is a potential interventional radioembolization candidate for HCC therapy.

### Supplementary Information

**Additional file 1: Supplementary Fig. 1.** (A). Hemolysis rate for PDA-CS. (B). Blood coagulation index for PDA-CS. (C). Cellular cytotoxicity for PDA-CS. (D). SEM image of PDA-CS in rat serum at 2, 4, 8, and 20 w.

**Additional file 2: Supplementary Fig. 2.** Blood examination of rats who received ^177^Lu-PDA-CS-MgO, ^177^Lu-PDA-CS microspheres or ^177^Lu-Lipiodol treatment, respectively. WBC, white blood cell; RBC, red blood cell; HGB, Hemoglobin; PLT, platelet.

## Data Availability

The datasets used and/or analyzed during the current study are available from the corresponding author on reasonable request.

## Abbreviations

HCC: Hepatocellular carcinoma
TACE: Transarterial chemoembolization
TARE: Transarterial radioembolization
FDA: Food and Drug Administration
SPECT: Single photon emission computed tomography
PDA: Polydopamine
MgO: Magnesium oxide
CS: Chitosan
DA: Dopamine
Span-80: Sorbitan monooleate
DEN: N-nitrosodiethylamine
HuVEC: Human umbilical vein endothelial cell line
FBS: Fetal bovine serum
FTIR: Fourier-transform infrared
TGA: Thermogravimetric Analysis
EDS: Energy dispersive spectroscopy
XPS: X-ray photoelectron spectroscopy
%ID/g: Injected dose per gram of tissue
RCS: Red cells suspension
BCI: Blood coagulation index
EANM: European Association of Nuclear Medicine
