# Supplementary figures and images for: Polymer composite microspheres loading 177Lu radionuclide for interventional radioembolization therapy and real-time SPECT imaging of hepatic cancer
Source: Biomater Res. 2023 Nov 4;27:110. doi: 10.1186/s40824-023-00455-x (PMC10625707; doi:10.1186/s40824-023-00455-x)

**
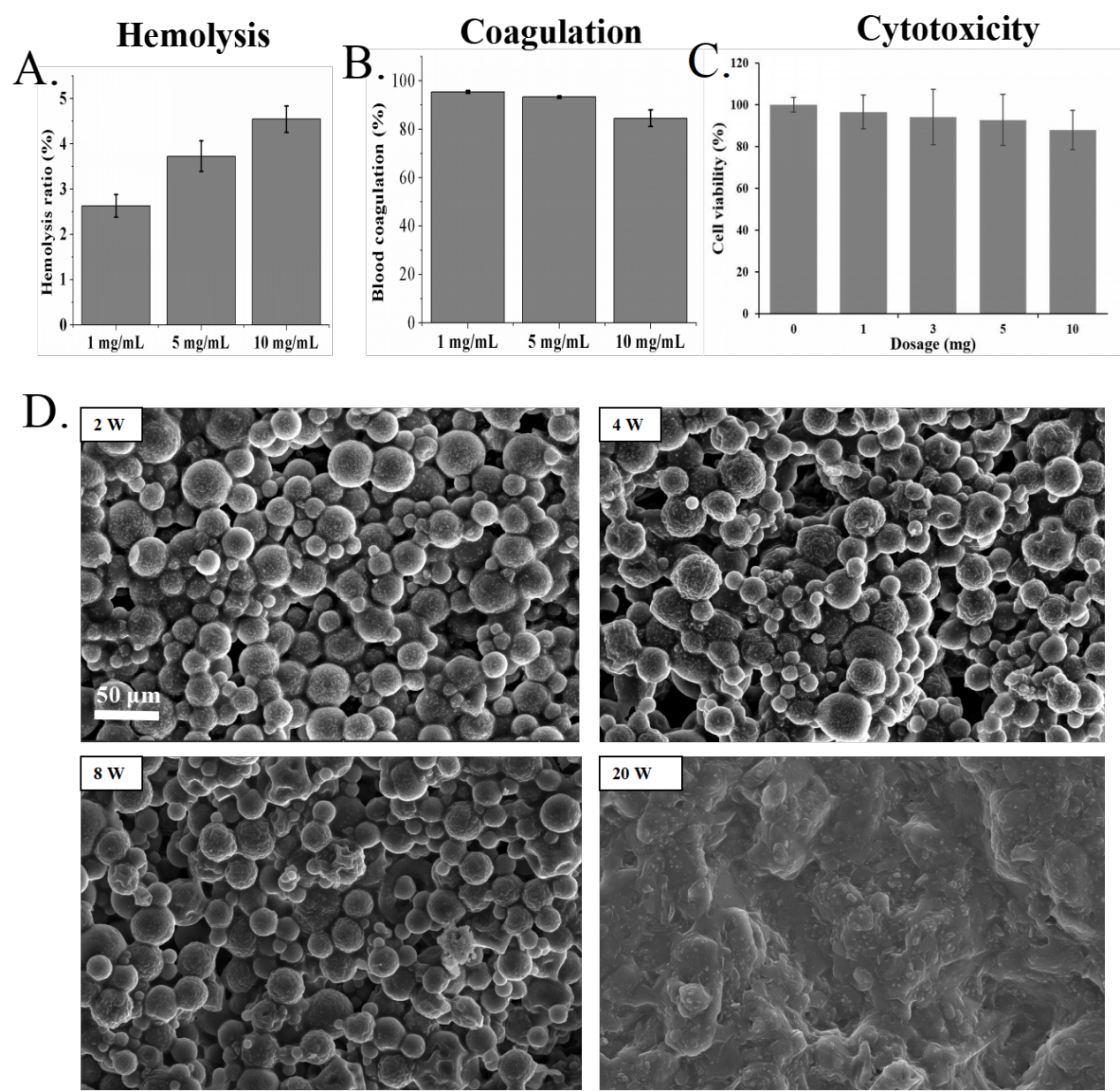
**

**Supplement figure 1.**

Supplement: Supplementary file 1 — Additional file 1: Supplementary Fig. 1. (A). Hemolysis rate for PDA-CS. (B). Blood coagulation index for PDA-CS. (C). Cellular cytotoxicity for PDA-CS. (D). SEM image of PDA-CS in rat serum at 2, 4, 8, and 20 w. [file 40824_2023_455_MOESM1_ESM.docx]

**
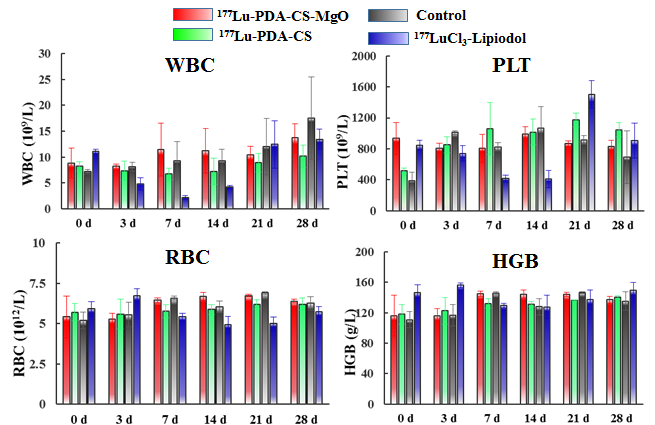
**

**Supplement Figure 2.**

Supplement: Supplementary file 2 — Additional file 2: Supplementary Fig. 2. Blood examination of rats who received 177Lu-PDA-CS-MgO, 177Lu-PDA-CS microspheres or 177Lu-Lipiodol treatment, respectively. WBC, white blood cell; RBC, red blood cell; HGB, Hemoglobin; PLT, platelet. [file 40824_2023_455_MOESM2_ESM.docx]
